# Modern imaging and image-guided treatments of the prostate gland: MR and ablation for cancer and prostatic artery embolization for benign prostatic hyperplasia

**DOI:** 10.1259/bjro.20190019

**Published:** 2019-08-14

**Authors:** João Lopes Dias, Tiago Bilhim

**Affiliations:** 1 Nova Medical School, Universidade Nova de Lisboa, Lisbon, Portugal; 2 Department of Radiology, Centro Hospitalar Universitário de Lisboa Central, Lisbon, Portugal; 3 Department of Radiology, Hospital Lusíadas, Lisbon, Portugal; 4 Interventional Radiology, Saint Louis Hospital, Lisbon, Portugal; 5 Interventional Radiology Unit, Centro Hospitalar Universitário de Lisboa Central, Lisbon, Portugal

## Abstract

Multiparametric MRI (mpMRI) has proven to be an essential tool for diagnosis, post-treatment follow-up, aggressiveness assessment, and active surveillance of prostate cancer. Currently, this imaging technique is part of the daily practice in many oncological centres. This manuscript aims to review the use of mpMRI in the set of prostatic diseases, either malignant or benign: mpMRI to detect and stage prostate cancer is discussed, as well as its use for active surveillance. Image-guided ablation techniques for prostate cancer are also reviewed. The need to establish minimum acceptable technical parameters for prostate mpMRI, standardize reports, uniform terminology for describing imaging findings, and develop assessment categories that differentiate levels of suspicion for clinically significant prostate cancer led to the development of the Prostate Imaging Reporting and Data System that is reviewed. Special focus will also be given on the most up-to-date evidence of prostatic artery embolization (PAE) for symptomatic benign prostatic hyperplasia (BPH). Management of patients with BPH, technical aspects of PAE, expected outcomes and level of evidence are reviewed with the most recent literature. PAE is a challenging technique that requires dedicated anatomical knowledge and comprehensive embolization skills. PAE has been shown to be an effective minimally-invasive treatment option for symptomatic BPH patients, that can be viewed between medical therapy and surgery. PAE may be a good option for symptomatic BPH patients that do not want to be operated and can obviate the need for prostatic surgery in up to 80% of treated patients.

## Introduction

More than doing an exhaustive theoretical exposition, the authors preferred to explore the advantages of multiparametric MRI (mpMRI) in the management of prostate cancer within different clinical scenarios that both radiologists and urologists may face. The most up-to-date evidence of prostatic artery embolization (PAE) for symptomatic benign prostatic hyperplasia (BPH) will be reviewed. Management of patients with BPH, technical aspects of PAE, expected outcomes and level of evidence are reviewed with the most recent literature.

## prostate cancer

### Multiparametric magnetic resonance imaging (mpMRI)

mpMRI of the prostate gland mpMRI combines morphological and functional sequences: axial, coronal, and sagittal high-resolution *T*_2_ weighted images (*T*_2_WI) are obtained to depict the zonal anatomy prostate and surrounding organs. An axial *T*_1_ weighted imaging (*T*_1_WI) sequence is typically obtained for whole pelvis analysis, allowing the identification of prostatic hemorrhage, abnormal lymph nodes, and suspicious bone lesions. These morphological sequences are combined with diffusion-weighted imaging (DWI) and dynamic contrast-enhanced (DCE) images, two functional sequences that increase both sensitivity and specificity on detection and staging of prostate cancer. MR spectroscopy is no longer routinely used in prostate mpMRI.^1–6^ Table 1 shows detailed technical parameters of our protocol on a 3 T magnet.

**Table 1.** Acquisition protocol on a 3T magnet

−30 min protocol (including preparation and positioning);
−without an ERC;
−18-channel PPA;
−antiperistaltic drugs (Buscopan^®^, Glucagon^®^).
−*T*_2_WI; axial, sagittal, and coronal; 3,5 mm, no gap; FOV 200 mm; matrix 384 × 384;
−DWI and ADC map; axial; 3,5 mm, no gap; FOV 200 mm; matrix 116 × 116; *b*-values: 0, 50, 1200, and 1400 s/mm^2^;
−DCE-MRI; axial; 3,5 mm, no gap; FOV 260 mm; matrix 154 × 192; maximum temporal resolution 15 s following single dose of contrast agent with an injection rate of 3 ml s^−1^; 30–35 acquisitions during 5 min.

ADC, apparent diffusion coefficient; DCE, dynamic contrast-enhanced; DWI, diffusion-weighted imaging; ERC, endorectal coil; FOV, field of view; PPA, pelvic phased arrays; *T*_2_WI, *T*_2_ weighted imaging.

The current use of high-quality flexible body coils and properly configured 3 T MRI systems makes endorectal coils unnecessary. Some disadvantages were recognized by many radiologists, including slowed workflow, increased patient discomfort, decreased patient adherence, and risk of rectal injury.^7^ The first version of Prostate Imaging Reporting and Data System (PI-RADS) was published by the European Society of Urogenital Radiology in 2012. In 2014 the second version (PI-RADSv 2) showed some upgrades that have been recently updated to PIRADSv. 2.1 ^8–10^ . Table 2 resumes PI-RADSv. 2.1 criteria.

**Table 2.** PI-RADSv. 2.1 criteria

**Findings in the peripheral and transition zones at DWI-ADC**
1. No abnormality (*i.e.* normal) on ADC maps and high *b* *-*value diffusion-weighted MR
2. Linear/wedge shaped hypointense on ADC and/or linear/wedge shaped hyperintense on high *b*-value DWI. Non-focal hypointense on ADC and/or hyperintense on high *b*-value DWI (for transition zone only)
3. Focal (discrete and different from the background) hypointense on ADC and/or focal hyperintense on high *b*-value DWI; may be markedly hypointense on ADC or markedly hyperintense on high *b*-value DWI, but not both.
4. Focal abnormality that is markedly hypointense on ADC maps and markedly hyperintense on high *b* *-*value diffusion-weighted MR images and measures less than 1.5 cm in greatest dimension
5. Same as score of 4, but abnormality measures 1.5 cm or more in greatest dimension or has definite extraprostatic extension or invasive behavior
**Appearance of peripheral zone abnormalities at *T*_2_ weighted MRI**
1. Uniformly hyperintense (normal)
2. Linear, wedge-shaped, or diffuse mild hypointensity, usually indistinct margin
3. Noncircumscribed, rounded, moderate hypointensity
4. Circumscribed, homogeneous moderately hypointense focus or mass confined to the prostate and less than 1.5 cm in greatest dimension
5. Same as score of 4, but abnormality measures 1.5 cm or more in greatest dimension or has definite extraprostatic extension or invasive behavior
**Appearance of transition zone abnormalities at *T*_2_ weighted MRI**
1. Homogeneous intermediate signal intensity (normal) or round, completely encapsulated nodule (“typical nodule”)
2. A mostly encapsulated nodule or a homogeneous circumscribed nodule without encapsulation (“atypical nodule”) or a homogeneous mildly hypointense area between nodules
3. Heterogeneous signal intensity with obscured margins
4. Lenticular or circumscribed, homogeneous, moderately hypointense, and less than 1.5 cm in greatest dimension
5. Same as score of 4, but abnormality measures 1.5 cm or more in greatest dimension or has definite extraprostatic extension or invasive behavior

ADC, apparent diffusion coefficient; DWI, diffusion-weighted imaging.

### 2.2. detection of prostate cancer

The traditional blind transrectal ultrasound (TRUS) biopsy aims to sample the peripheral zone, where the majority of cancers arise. In many urological centers, TRUS biopsy gets two cores per sextant that may miss small, aggressive peripheral cancers, as well as transitional, central or fibromuscular tumors. On the other hand, non-significant peripheral tumors may be diagnosed, thus leading to unnecessary treatments.^11–14^ Screening strategies involving the use of mpMRI rather than TRUS biopsies have shown higher sensitivity and specificity in the diagnosis of prostate cancer. Moreover, patient morbidity showed an apparent significant decrease, not only during diagnosis—avoiding unnecessary repeated biopsies—but also while choosing treatment—reducing overtreatment in low-risk cancers.^15^ One of the main advantages of mpMRI is then to identify suspicious lesions that might undergo guided biopsy. Since 40–60% of prostate tumors are invisible at the ultrasound, MRI guidance should also be considered for a more accurate biopsy. It may be performed either directly (in-bore biopsy) or indirectly (using fusion techniques that overlap TRUS images and previously obtained MR images).^16–20^

**Figure 1.**
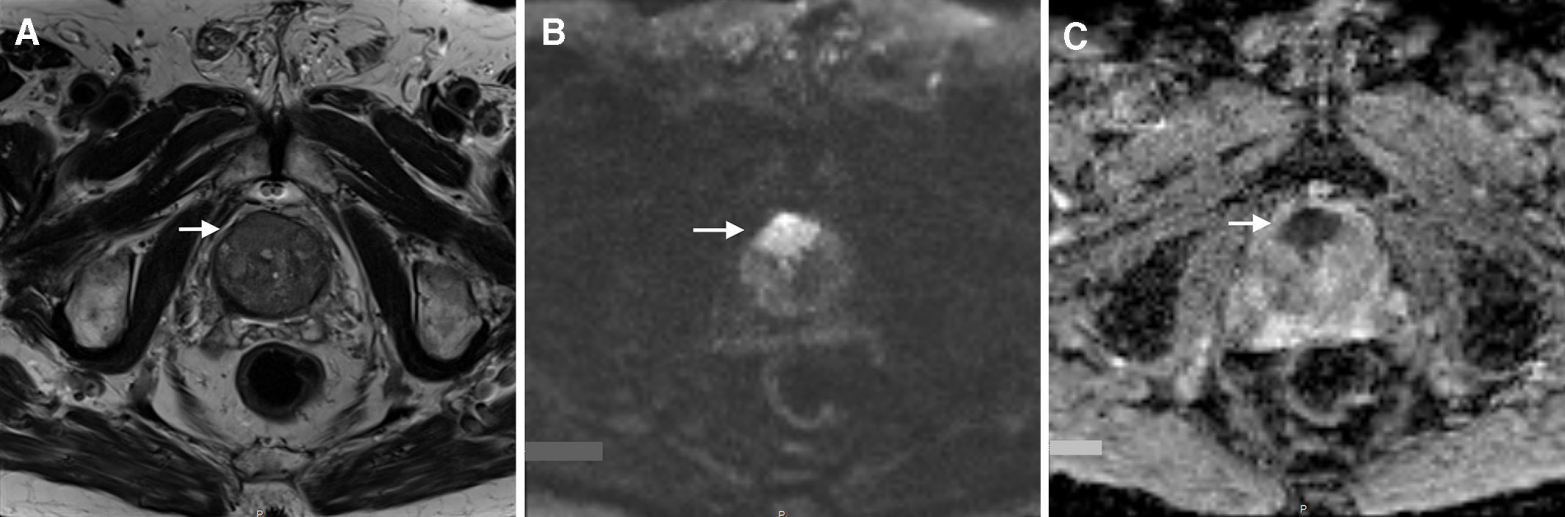
Axial images of a 75-year-old male with an elevated PSA level (19 ng ml^−1^) who had undergone three previous negative systematic TRUS-guided biopsies. A huge bilateral, irregularly shaped focal lesion is seen in the anterior transition and zone anterior fibromuscular stroma (white arrows), moderately hypointense on *T*_2_WI (a), very restrictive at high *b* *-*value (b) and dark on the ADC map (c). Because of its size (>15 mm), this lesion was assigned a PI-RADS 5. A MRI/ultrasound fusion-guided biopsy was performed and an adenocarcinoma, Gleason score of 8 (4 + 4), was diagnosed. ADC, apparent diffusion coefficient; *T*_2_WI,*T*_2_ weighted imaging; PI-RADS, Prostate Imaging Reporting and Data System; PSA, prostate-specificantigen; TRUS, traditional blind transrectal ultrasound.

In the set of cancer detection, mpMRI reports should always include PI-RADS classification. PI-RADS categories 1 and 2 refer to normal and benign changes, respectively. On the other hand, in categories 4 and 5, clinically significant cancer is likely and highly likely to be present, respectively (Figure 1). A score of 3 includes indeterminate, equivocal findings. According to PI-RADSv. 2.1, the score assigned on the ADC map and at DWI with high *b* *-*values is the dominant parameter for peripheral zone lesions. However, for abnormalities in the transition zone, the evaluation is based primarily on the score assigned at *T*_2_WI. DCE sequences have now more limited importance in the overall assessment of the prostate and only remain useful in the evaluation of peripheral lesions that were given a score of 3 on DWI/ADC analysis. Those lesions will be considered positive at DCE-MRI if a focal abnormality with early and intense enhancement is found, thus increasing the overall PI-RADS score from 3 to 4.^21,22^

### staging

**Figure 2.**
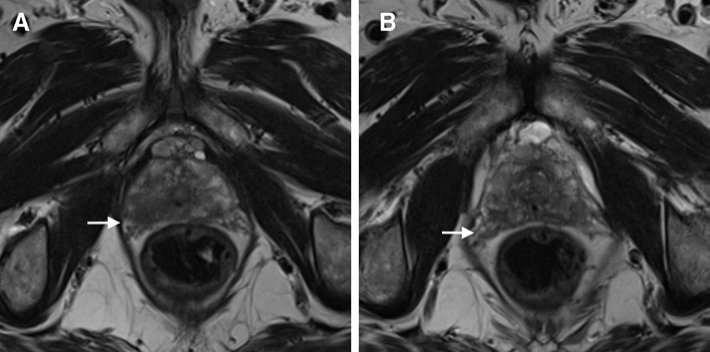
*T*_2_W axial images of a 63-year-old male with an elevated PSA level (8,4 ng ml^−1^). An ill-defined, hypointense lesion is seen in the right peripheral zone (white arrows). Local bulging and irregularity of the capsule are also seen, as well as thickening of neurovascular bundle (clearly depicted in comparison to the opposite side). Due to extracapsular extension (including neurovascular bundle invasion), this lesion was assigned a PI-RADS 5. A MRI/ultrasound fusion-guided biopsy was performed and an adenocarcinoma, Gleason score of 8 (4 + 4), was diagnosed. PI-RADS, Prostate Imaging Reporting and Data System; PSA, prostate-specific antigen; *T*_2_W, *T*_2_ weighted.

The eighth TNM edition introduced some important exchanges on prostate cancer. Pathological organ-confined disease (after radical prostatectomy)—pT2—is no longer subdivided into pT2a, pT2b, or pT2c. However, the extent of involvement and laterality remain important topics to report in mpMRI since the evolution from whole-gland to minimally invasive focal therapies depends on a more accurate location of prostatic tumors.^23^ pT3a still refers to tumors with extra capsular extension (ECE) or microscopic invasion of the bladder neck. While ECE may be identified on staging MRI, microscopic invasion of the bladder neck is below its visibility threshold. Multiple criteria are used to detect ECE, including capsule bulging, obliteration of the rectoprostatic angle, asymmetry of the neurovascular bundle, focal capsular retraction and or thickening, tumor/capsule contact greater than 15 mm, or the presence of tumoral nodules in fat surrounding tissues. Despite not being specifically included in the TNM system, the evaluation of neurovascular bundles is another key point for patients who are candidates to nerve-sparing surgeries (like robotic-assisted radical laparoscopic prostatectomy) (Figure 2). DWI and DCE increase the accuracy of MRI in the depiction of ECE (and more precisely neurovascular bundle invasion) once truthful protocols are used.^24–29^ T3b refers to the invasion of the seminal vesicles, typically seen as *T*_2_WI hypointense lesions, restrictive pattern on DWI and enhancement on DCE images. Other findings like enlarged low-signal-intensity ejaculatory ducts and obliteration of the angle between the prostate and the seminal vesicle may be also seen.^23^

Nodal staging is also performed in routine mpMRI protocols. This remains a challenging and misunderstood field for radiologists since morphological and dimensional criteria still seem to be not enough. The utility of DWI for nodal staging needs more research and may be time-consuming in daily practice.^30^ Routine mpMRI protocols do not allow an accurate evaluation of bone, not only due to the lack of morphological sequences like fat saturated *T*_2_WI or short tau inversion-recovery but also because the entire skeleton is not within the range of study. Whole body MRI protocols have been developed over the last years and seem to be very useful for bone staging in prostate cancer, achieving similar results when comparing to bone scintigraphy and other nuclear studies.^31^

### Active surveillance

Active surveillance is an alternative management for patients with a low clinical stage based on digital rectal examination (DRE) of the prostate, favorable prostate-specific antigen (PSA) levels evolution, low biopsy tumor grade, and minor tumor volume. Strict guidelines are still missing and reported inclusion criteria vary. Most urological departments consider active surveillance in males with low-risk non-metastatic prostate cancer (cT1–cT2; PSA <10 ng ml^−1^; and biopsy Gleason score of 6).^32–38^ mpMRI can be used to detect clinically significant prostate cancer in males on active surveillance. Specific criteria for progression are still requiring but some theoretical concepts may be applied, namely size increase of the index lesion, onset of other suspicious lesions and new signs of locally invasive disease. It is theoretically expectable that a decrease in ADC values within a lesion between two examinations corresponds to a histological upgrade of the tumor. However, the noted variability of ADC quantification across imaging platforms and institutions, as well as across different examinations in the same patient even within normal tissue (*e.g.* due to different positioning of the region of interest), hampers this evaluation. Thus, radiologists should verify the accuracy of quantitative ADC measurements on their own MR system (*e.g.* using phantoms) and compare their results with those derived from similar MR systems.^36–42^ Overall, it is clear that mpMRI is useful for monitoring of males on active surveillance, but there is not enough evidence that it may replace the repetition of standard biopsy to detect progression over time.^36,37,43^

### Ablation procedures

Besides the lack of ionizing radiation, interventional MRI (iMRI) offers other interesting advantages: the capability to guide needles and devices in any orientation; the inherent high tissue contrast; the ability to measure tissue firmness and thermal distribution through modern sequences; the development of real-time sequences. However, some disadvantages should not be forgotten, like the need for expensive nonferromagnetic tools and the small apertures and long bores that are typically found in the majority of MRI systems.^44,45^ Some focal therapies may use MRI as a guiding-method, such as focal laser ablation, cryoablation, and high-intensity focused ultrasound (HIFU). These techniques are clinically safe and effective, with few associated sexual and urinary side-effects. Despite being more commonly used in the initial treatment of prostate cancer, they may be theoretically performed in post-surgical and post-RT local recurrences, whenever it is accessible.^44^ More specific technical details about each treatment are beyond the scope of this article.

### Biochemical failure

PSA level remains the basis of follow-up after curative treatment, but the definition of biochemical failure differs between RP and external beam radiotherapy (EBRT). After RP, it is defined by two consecutive PSA values of >0.2 ng ml^−1^. After EBRT, with or without short-term hormonal manipulation, it is defined by a PSA increase >2 ng ml^−1^ higher than the initial PSA nadir value.^46,47^

**Figure 3.**
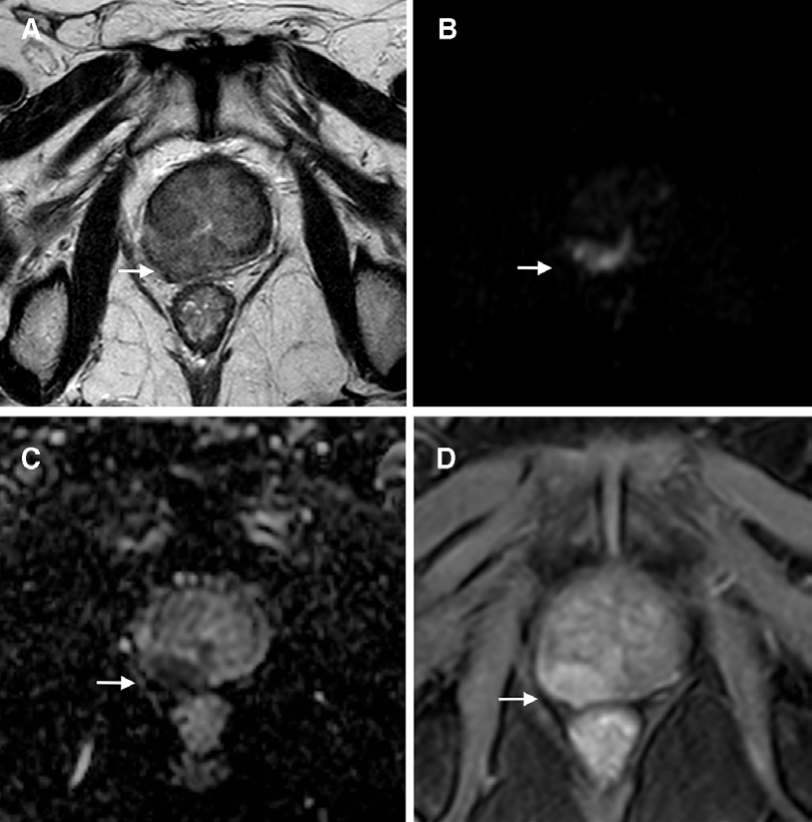
Axial images of a 66-year-old male with an elevated PSA level (5,3 ng ml^−1^) who had undergone EBRT 6 years before because of an adenocarcinoma of the prostate, Gleason score of 6 (3 + 3). An ill-defined lesion is seen in the right peripheral zone (white arrows), moderately hypointense on *T*_2_WI (a), very restrictive at high *b*-value (b), dark on the ADC map (c), and enhancing on DCE images (d). Due to local capsular irregularity, this lesion was assigned a PI-RADS 5. A MRI/ultrasound fusion-guided biopsy was performed and an adenocarcinoma, Gleason score of 7 (4 + 3), was diagnosed. ADC, apparent diffusion coefficient; DCE, dynamiccontrast-enhanced; EBRT, external beam radiotherapy; PI-RADS,Prostate Imaging Reporting and Data System; PSA, prostate-specific antigen; *T*_2_WI, *T*_2_ weightedimaging.

Local relapse after RP commonly appears in the retrovesical space and at the vesicourethral anastomosis. It is usually seen as an interruption of the normal hypointense ring of the bladder neck and vesicourethral anastomosis by a tumor with higher signal intensity on *T*_2_WI. Moreover, recurrence typically presents as early enhancing nodules on DCE images and as bright foci on DWI at high *b*-values. In these cases, current guidelines do not contemplate PI-RADS classification.^47,48^ After EBRT, fibrosis and changes on parenchymal vascularization modify the biological behavior of tumors and normal tissue. Typical zonal anatomy of the prostate is lost and diffuse low signal intensity is seen on *T*_2_WI, thus diminishing the contrast between tumor and irradiated tissue, which hampers recurrence detection on morphological sequences. Despite also being distorted by EBRT, DCE-MRI and DWI are more accurate than *T*_2_WI in the depiction of parenchymal recurrence (Figure 3).^47^

There is no consensus on biochemical failure after transperineal brachytherapy and other less invasive alternative treatment options like cryosurgery and HIFU. Recurrence semiology is similar to that of post-EBRT studies.^47^ After non-surgical therapies like EBRT, brachytherapy, cryosurgery or HIFU, PI-RADS classification may be used and a guided biopsy may be performed if a suspicious lesion is found. Since post-treatment changes may mimic tumors, essentially on morphological sequences, it is not surprising if the number of PI-RADS 3 is higher than desirable.

## Prostatic artery embolization for benign prostatic hyperplasia

### Patient management

Even though BPH is a histologic diagnosis, most patients are diagnosed based on the presence of lower urinary tract symptoms (LUTS) and benign prostatic enlargement (BPE). Biopsy is usually performed to exclude malignancy as histologic findings of BPH are ubiquitous in the older male. BPE may cause bladder outlet obstruction (BOO) and LUTS. Multidisciplinary evaluation of patients with symptomatic BPH is paramount as clinical management of these patients may be complex. LUTS may be caused by many other etiologies as bladder dysfunction, bladder neck contractures, nocturnal polyuria, urethral stricture, malignancy and nervous system dysfunction amongst others. The presence and severity of LUTS do not correlate with the presence of BOO or the size of the prostate. Thus, a patient may have BPH and have no LUTS or BOO. The severity of BOO should be evaluated with peak urinary flowrate (Qmax) and post-void residual volume (PVR) measurements in all patients with LUTS because it may lead to complications such as recurrent urinary tract infections, bladder stones, overflow incontinence, gross hematuria, hydronephrosis, acute urinary retention, and renal disease that prompt invasive treatment.^49,50^ However, LUTS and prostatic volume (PV) are the main drivers for seeking medical care, as BOO frequently does not worry the patients. When dealing with these patients there are three main goals: reduce LUTS, decrease PV and relieve BOO severity in order to provide symptom control and possibly prevent disease progression.

The presence and severity of LUTS should be quantified with validated questionnaires such as the international prostate symptom score (IPSS) that is a powerful tool to assess treatment response. The IPSS score has an additional question regarding the quality of life (QoL) related to the LUTS that is very important as this is the main driver to opt for more invasive treatment approaches when QoL >4 points. The IPSS score does not diagnose the presence or severity of BOO, nor the cause of LUTS. Thus, it is important to exclude all potential non-prostatic causes of LUTS before invasive treatment options are pursued. It is also important to assess the presence and severity of BOO with uroflowmetry and bladder ultrasound to assess the Qmax and PVR. BPE should be accurately quantified with transrectal ultrasound and/or MR imaging to assess the PV reduction 1 month, 6 months, 12 months and then yearly after intervention. In order to exclude malignancy, digital rectal examination (DRE), PSA and mpMRI should be performed and biopsy if PSA >4 ng ml^−1^ and/or suspicious DRE/mpMR. Erectile and ejaculatory function and incontinence severity index (ISI) should be evaluated before and after prostatic interventions. Validated questionnaires as the international index of erectile function (IIEF) and the male sexual health questionnaire-ejaculation disorder (MSHQ-EjD) are proven useful tools.

Patient age, PV, IPSS/QoL, Qmax, PVR and PSA should all be considered when counseling patients with LUTS for PAE. Inclusion criteria are: age above 40 years, PV greater than 40 cm^3^, IPSS >18 points and/or QoL >3 points, Qmax < 12 ml s^−1^, PVR <200 cm^3^ and PSA <4 ng ml^−1^. If Qmax > 12 ml s^−1^ and/or PVR >200 cm^3^, when there is clinical suspicion of neurological disease or diabetes, patients under 40 years of age or PV <40 cm^3^, invasive urodynamic testing should be performed to exclude non-prostatic causes of LUTS. Exclusion criteria include: malignancy, large bladder stones (>4 cm) or diverticula (>5 cm), advanced atherosclerosis (severe stenosis or occlusion) and tortuosity (more than two angulations < 90°) of iliac arteries or non-visualization of prostatic arteries (PAs) on pre-procedural CT angiography (CTA), urinary tract infection or renal insufficiency due to prostatic obstruction. Medical therapy is a first-line treatment option before considering invasive treatments for LUTS and BPH. Generally, PAE may be considered for those patients refractory to medical therapy for more than 6 months, not wishing or not tolerating medical therapy. Acute urinary retention (AUR) is also a good indication for PAE, as these patients have very good outcomes with embolization. For AUR patients, only PV and PSA should be measured as the IPSS/QoL, IIEF, MSHQ-EjD or ISI scores and Qmax/PVR are not reliable.

### Technical aspects

PAE is a challenging technique due to two main reasons: complex anatomy and tortuosity of the PAs that may be very challenging to selective catheterize. PAs are small, lack pathognomonic findings and the internal iliac artery has many other side branches. Thus, one can easily “get lost” inside the pelvic arteries looking for the PAs.^51^ The tortuous pelvic arterial anatomy and the presence of atherosclerosis in aging male patients are the main reasons for PAE being successful in only one pelvic side (unilateral PAE) in up to 15% of patients.^52^ Two or more PAs can be found in up to 40% of pelvic sides with the most frequent origins being the superior vesical artery (30%), the internal pudendal artery (30%), the anterior division of the internal iliac artery (15%) and the obturator artery (10%). Rare/variant PA origins in up to 15% of pelvic sides include: accessory pudendal arteries (2%), prostatorectal trunks (7%), superior gluteal (1%) and inferior gluteal arteries (4%), aberrant obturators (1%) and penile artery (<1%). In up to 60% of pelvic sides, anastomoses can be detected between the prostatic arterial bed and the surrounding organs including the penis, rectum, bladder and seminal vesicles that may lead to non-target embolization.^51,53,54^ To overcome these challenges, steep ipsilateral oblique views have been adopted for PAE procedures^51,54^ that may lead to high radiation exposure to both patients and interventionalists.^55^

**Figure 4.**
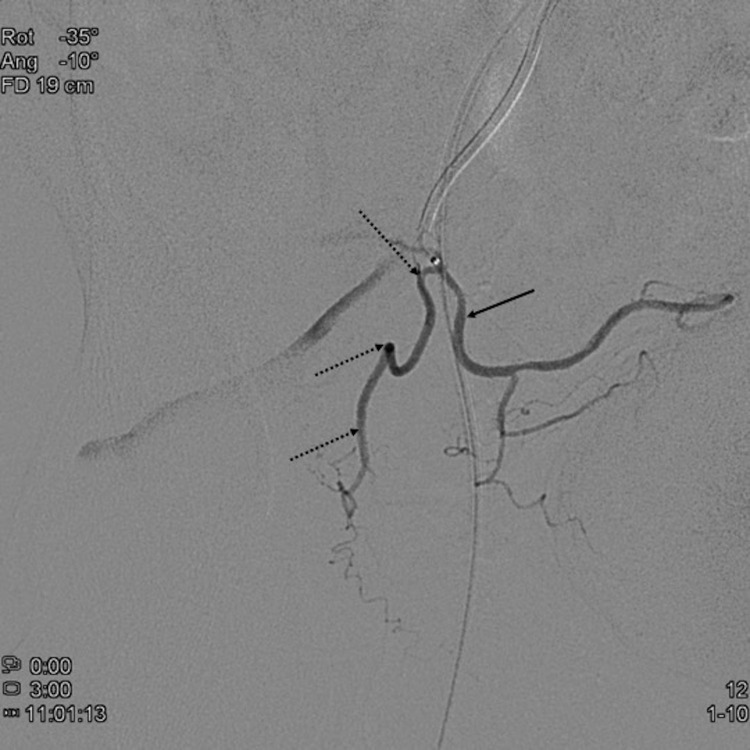
Right anterior oblique view digital subtraction angiogram showing the right prostatic artery (dashed arrows) arising from the superior vesical artery (arrow). Note the acute angulation of the prostatic origin.

**Figure 5.**
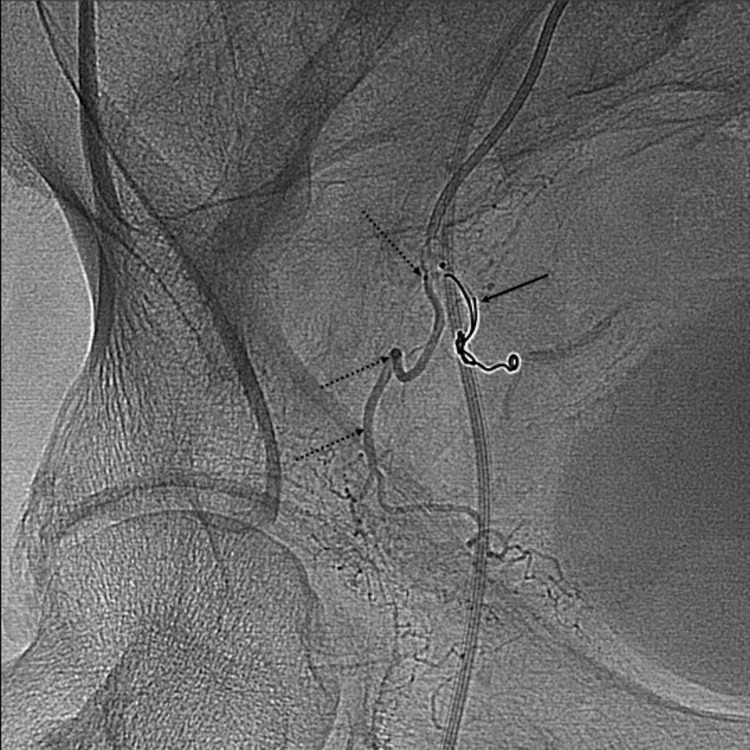
Selective catheterization of the prostatic artery was not possible due to the acute angulation of the origin. Thus, coil placement was performed in the superior vesical artery (arrow) to redirect flow away from the bladder and into the prostatic artery (dashed arrows).

**Figure 6.**
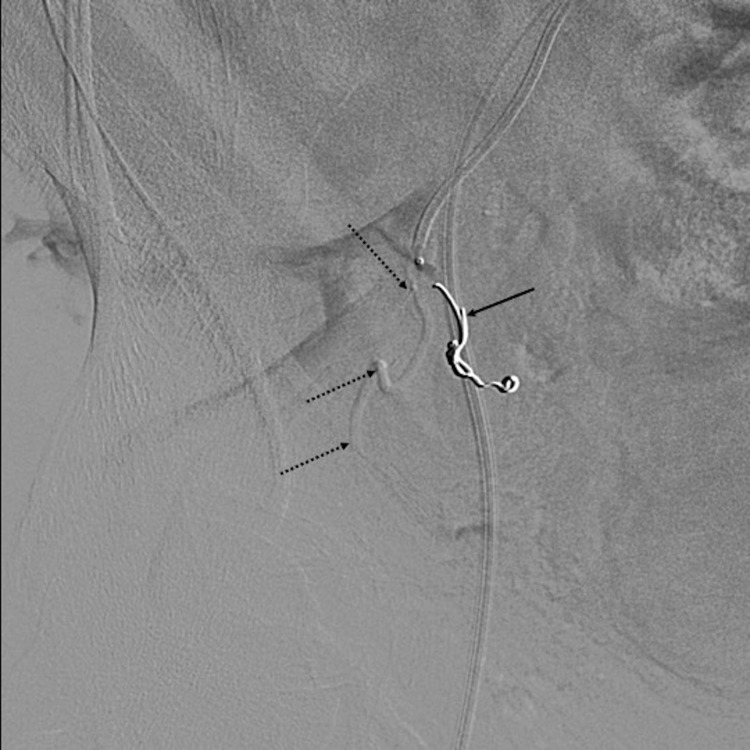
Control angiogram after embolization of the prostatic artery shows stasis in the prostatic artery (dashed arrows) and the protective coil in the superior vesical artery (arrow).

**Figure 7.**
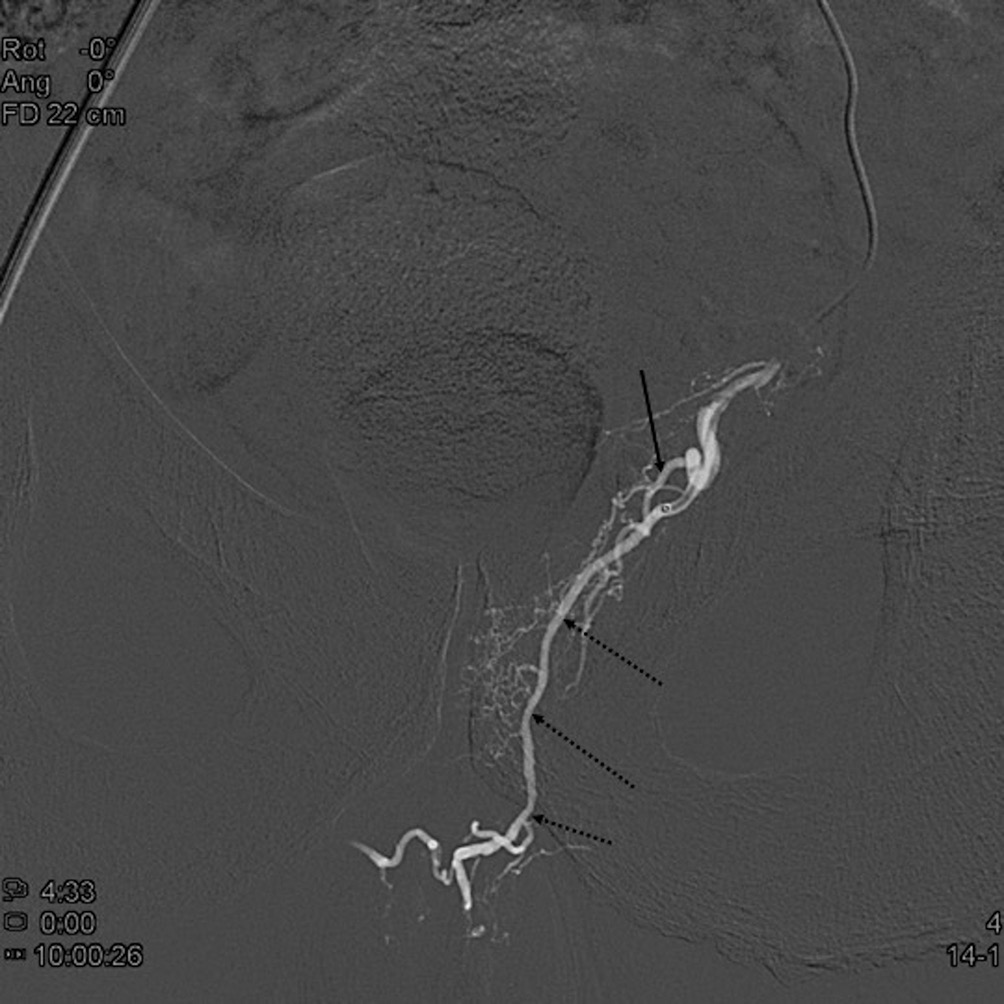
Selective angiogram of the left prostatic artery (arrow) in posteroanterior view showing a large anastomosis to the penile artery (dashed arrows).

**Figure 8.**
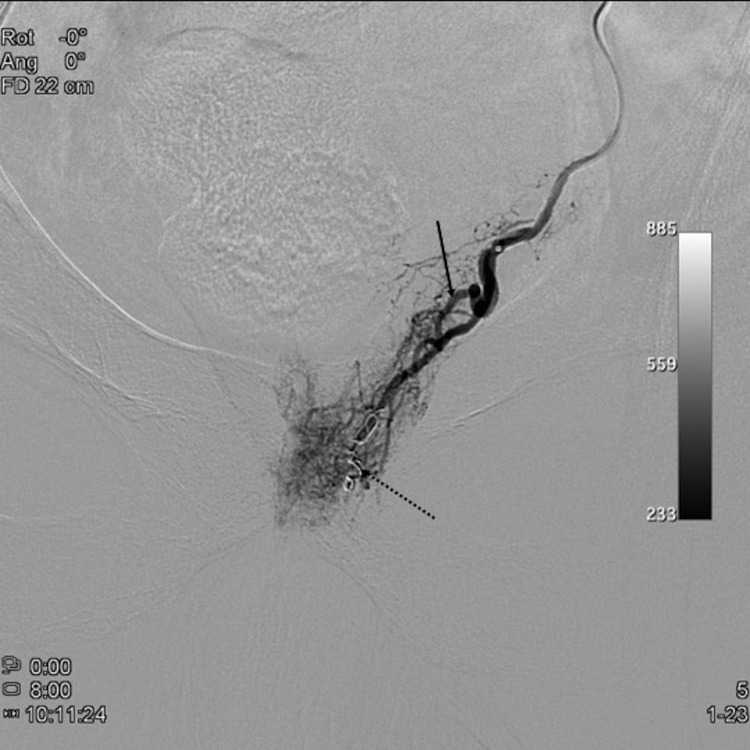
Selective angiogram of the left prostatic artery (arrow) in posteroanterior view after coil blockage of the penile anastomosis (dashed arrow) depicting prostatic central gland opacification and no penile branches.

Either pre-procedural CTA^51^ or intra procedural cone beam CT (CBCT)^56,57^ should be used to guide interventionalists during PAE. Both techniques are equally effective for identifying the PAs.^58^ CBCT can also be used to certify correct catheterization of the PAs and exclude anastomoses that may lead to non-target embolization.^56,57^ CTA, even with optimized protocols using sublingual glyceryl trinitrate^59^ fails to identify most anastomoses. CBCT data can also be used for automatic vessel detection analysis, obviating the need for extensive anatomical knowledge.^60^ MRA has been recently validated to define the anatomy of the PAS with a sensitivity of 92%.^61^ When anastomoses to surrounding organs are identified, coil occlusion has been shown to be safe and effective to prevent non-target embolization^62^ or to redirect flow into the prostate (Figures 4–8). Relevant variants include accessory pudendal arteries, prostatorectal trunks and aberrant obturators that may be identified with pre-procedural CTA.^51^ Besides femoral access, radial access has also been shown to be safe and effective for PAE.^62^

The use of pre-shaped Swan-neck tip microcatheters ≤ 2.4 F are the first-line option for many experienced interventionalists in PAE. The vascular access is usually 5 F and the catheters used vary considerably. When using a femoral approach, the cross-over technique will be necessary and the Waltman loop frequently performed to allow bilateral embolization through a single femoral access. Regarding embolic preference, microspheres 100–500 µm^63^ and PVA particles 100–300 µm^64^ have been shown to be safe and effective. The use of microspheres <300 µm has the potential to induce more adverse events and untargeted embolization with limited clinical benefit.^63,65,66^ We advise using microspheres >300 µm or PVA particles 100–300 µm independently of prostate volume or presence of collaterals. No specific embolic agent has proven superiority.^63–66^ We use of 100–200 µg of intra-arterial nitroglycerin inside the PAs just before the start of embolization to avoid spasm and early stasis.

### Expected outcomes

The main goals of PAE are: 1. LUTS relief; 2. PV reduction; 3. Relief of BOO with increase in Qmax and PVR reduction. Also, adverse events should be prospectively registered and classified alongside with the collection of the IIEF, MSHQ-EjD and ISI scores. The need for prostatic medication or surgery after PAE should also be assessed with long-term data. Meta-analyses have proved significant treatment efficacy of PAE with IPSS reduction from baseline of 12.93, 14.98, 15.00 points at 1, 3 and 6 months post-PAE (50%–70% symptomatic relief); QoL reduction from baseline of 2.17, 2.18, 2.15 and 2.49 points at 1, 3, 6 and 12 months post-PAE.^67^ Mean PV reduction after PAE is approximately 15–30%, with changes from baseline of 15–30 cm^3^ at 1, 3, 6 and 12 months post-PAE.^67^ The Qmax increase after PAE is approximately 4.66 to 5.82 mL s^−1^ at 1, 3, 6 and 12 months post-PAE (20%–50% increase) and the PVR decrease after PAE is approximately 62*–*85 mL at 1, 3, 6 and 12 months post-PAE.^67^ PSA has also been shown to reduce significantly after PAE with mean decrease of 1.2 ng ml^−1^ (15%–20%). IIEF scores after PAE did not show significant changes from baseline,^67^ ejaculation has also been prospectively shown to be preserved after PAE^68^ and no reports of incontinence after PAE exist so far.

PAE is a very well-tolerated procedure with almost no pain and no nausea or vomiting associated. It is an ideal procedure to be performed on an outpatient setting with the main concern being the arterial access hemostasis that may be obliviated with the use of radial access or femoral closure devices. The overall adverse events rate of PAE is expected to be 33%, with the vast majority (99%) being minor complications that need neither specific medical or surgical treatment nor admission to the hospital. Most of these adverse events include the so-called post-PAE syndrome with frequency and burning sensation in the urethra in up to 40% of patients. Macroscopic hematuria may be present in 5.6%, hematospermia in 0.5% and rectal bleeding in 2.5% of patients in the first week post-PAE. These are the main adverse events after PAE and subside with conservative measures 1*–*2 weeks after PAE. Urinary tract infection has been reported in up to 7.6% of patients and AUR in 2.5% of patients after PAE.^69^ Some patients (<3%) may experience central gland detachment with prostatic fragments inside the urethra or bladder that may prompt removal by cystoscopy.^69^ Major adverse events that have been reported include a case of radiation dermatitis and those related to non-target embolization (<3%) to the penis, bladder, rectum and seminal vesicles.^67,69^

Most clinical failures after PAE are patients that never responded to embolization (non-responders)—up to 80% of all patients, with the remainder 20% being patients that initially improved, but then had relapsing symptoms (relapsers).^70^ Therefore, it is important to identify patient/baseline predictors as well as technical predictors of clinical success to help select the best candidates for PAE and exclude those with a low probability of success after PAE. Other useful tools to help predict treatment response include MRI, peak PSA and C-reactive protein values 24 h after PAE and contrast-enhanced ultrasonography.^70–77^ Younger patients (up to 65 years of age) with acute urinary retention and baseline IPSS of less than 23 points have been identified as responding better to PAE.^70^ Baseline prostate volume has yielded conflicting results with some studies showing better outcomes with larger prostates as opposed to others failing to prove any advantage.^69–80^ A cut-off of minimum PV for PAE has been shown to have implications in treatment outcomes—patients with PV <40 cm^3^ have been shown to have significantly worse outcomes after PAE.^75^ Patients with PV increase due to a large proportional increase of the central gland have been shown to respond better to PAE.^75^ Also, patient with multiple large (>1 cm) adenomas in the central gland have been shown to respond better to PAE than those without adenomas. The median lobe and central gland of the prostate are the regions of the prostate that respond better to embolization with higher areas of ischemia and larger volume reductions.^72^ Quantification parameters with mpMR including perfusion and diffusion parameters have failed to predict treatment outcomes after PAE.^76^ However, the proportion and the volume of prostate ischemia measured with MR in the first month after PAE have been shown to correlate significantly with the PSA level 24 h after PAE and with clinical outcome.^70^ MR after PAE should be performed within the first month post-PAE as ischemia usually disappears afterwards. No studies to date have focused in comparing the MR features between unilateral and bilateral PAE. High C-reactive protein values 24 h after PAE have also been shown to predict a good clinical outcome.^75^ The technique of PAE also has impact on outcomes: bilateral PAE has better outcomes than unilateral PAE.^52,70,77^ Particle size is a debatable issue^80^ with some studies failing to prove better outcomes with smaller (<300 µm) microspheres,^63,65^ whereas others have reported better outcomes.^75,77^ Predictors of technical difficulty include older age, atherosclerosis, tortuosity of pelvic vessels and PAs originating from the superior vesical artery.^77,81^ The interventionalist and the use of protective coil embolization have also been shown to significantly influence procedural times and radiation dose.^77^

### Evidence

Table 3 shows some of the most relevant Phase II trials that have proved PAE to be safe and effective.^68,82–93^ The comparative trials of PAE and surgery^94–98^ are presented in Table 4. The prospective comparative trials comparing PAE and surgery have shown that LUTS relief is similar between the two techniques which has led to widespread adoption in the UK.^99^ However, the relief of BOO and PV reduction is more effective with prostatic surgery than PAE. The rate of adverse events is lower with PAE, and surgery induces a higher rate of multiple adverse events and more serious adverse events. Recovery from intervention is faster with PAE than with surgery. All of these aspects should be taken into consideration when counseling patients with LUTS for PAE. PAE is effective at relieving LUTS and may be a viable option for patients after failed medication therapy or not tolerating prostatic medication. PAE does not preclude future prostatic surgeries that can always be performed in case the patient does not improve or when severe BOO is still present after embolization. PAE is an effective minimally invasive treatment option for patients with LUTS that can be viewed between medical therapy and surgery. General adoption from most urological societies is still lacking, as PAE is viewed as an experimental technique needing more long-term data and comparative trials.^50^ PAE may be a good option for patients with LUTS that do not want to be operated and can obviate the need for prostatic surgery in up to 80% of treated patients.

**Table 3.** Prospective Phase II trials of PAE for BPH

Study	Year	Country	N.pts	F.U. (months)	IPSS/QoL reduction	PV reduction	Qmax increase	PVR reduction	PSA reduction	Minor AE	Major AE	Other remarks
Salem et al. Urology	2018	USA	45	12	11.2/2.2 points	18%	9.5 ml s^−1^	48 ml	NR	58%	0%	
Bagla et al. JVIR	2014	USA	20	6	9.8/2.6 points	18%	NR	NR	NR	42%	0%	
Kurbatov et al. Urology	2014	Russia	88	12	13.6/3.3 points	58 ml (45%)	9.6 ml s^−1^	56.9 ml	NR	0%	0%	PV >80 ml
Grosso et al. Radiol Med	2015	Italy	13	8	17.1/2.6 points	28%	NR	NR	39.4%	0%	0%	
De Assis et al. CVIR	2015	Brasil	35	3	15.6/3.9 points	43 ml (32%)	8.1 ml s^−1^	NR	4.7 ng ml^−1^ (53%)	17.7%	2.9%	PV >90 ml
Wang et al. BMC Urol	2015	China	117	24	17/2 points	49 ml (42%)	6 ml s^−1^	85 ml	0.3 ng ml^−1^ (8%)	75%	0%	PV >80 ml
Isaacson et al. JVIR	2016	USA	12	3	18.3/3.6	34.4 ml (31%)	7.1 ml s^−1^	46.3 ml	NR	42%	0%	PV >80 ml
Pisco et al. JVIR	2016	Portugal	630	78	11.7/1.8	15 ml (19%)	3.3 ml s^−1^	44.8 ml	1.2 ng ml^−1^ (23%)	25%	0.4%	
Rampoldi et al. CVIR	2017	Italy	43	13	7.1/3.6	13 ml (20%)	NR	NR	NR	22%	0%	Poor surgical candidates
Bhatia et al. JVIR	2018	USA	93	12	15/3.1	43.6 ml (31%)	5.1 ml s^−1^	136 ml	3.5 ng ml^−1^ (46%)	43%	1.1%	PV >80 ml
Brown et al. BJU Int	2018	Australia	51	18	18.8/3.8	37 ml (32.3%)	2.1 ml s^−1^	42 ml	NR	84.3%	0%	
Tapping et al. CVIR	2018	UK	12	18	15/NR	NR	NR	NR	NR	0%	0%	Patients with haematuria
Overall (means)			1159	16	14/3	37 ml (29%)	6.4 ml s^−1^	66 ml	34%			

AE, adverse events; F.U., follow-up time in months; IPSS/QoL, international prostate symptom score/quality of life; NR, not reported; N. pts, number of patients; PSA, prostate-specific antigen; PV, prostatic volume; PVR, post-void residual volume; Qmax, peak urinary flowrate; UK, United Kingdom; USA, United States of America.

**Table 4.** Comparative studies of PAE *vs* prostatic surgery

Study	Year	Country	N.pts PAE/surgery	F.U (months)	IPSS/QoL changes between groups	PV reduction between groups	Qmax increase between groups	PVR reduction between groups	PSA reduction between groups	Minor AE	Major AE	Other remarks
Gao et al. Radiology	2014	China	57/57	24	Similar	TURP better	Similar	Similar	TURP better	Similar	Similar	RCT / TURP
Russo et al. Urology	2015	Russia	80/80	12	OP better	OP better	OP better	OP better	OP better	PAE better	PAE better	PSM / OP
Carnevale et al. CVIR	2016	Brasil	15/15	12	Similar	TURP better	TURP better	Similar	Similar	PAE better	PAE better	Prospective / TURP
Abt et al. BMJ	2018	Switzerland	48/51	3	Similar	TURP better	TURP better	TURP better	Similar	PAE better	PAE better	RCT / TURP
Ray et al. BJU Int	2018	UK	216/89	12	TURP better	TURP better	TURP better	TURP better	TURP better	PAE better	PAE better	PSM / TURP

AE, adverse events; F.U., follow-up time in months; IPSS/QoL, international prostate symptom score/quality of life; N. pts, number of patients; OP, open prostatectomy; PAE, prostatic artery embolization; PSA, prostate-specific antigen; PSM, propensity score matching (retrospective); PV, prostatic volume; PVR, post-void residual volume; Qmax, peak urinary flowrate; RCT, randomized controlled trial; TURP, trans-urethral resection of the prostate; UK, United Kingdom.
